# An Emerging Trend of Methamphetamine Injection in Iran: A Critical Target for Research on Blood-Borne Infection Diseases

**DOI:** 10.5812/hepatmon.8154

**Published:** 2013-02-28

**Authors:** Zahra Alam Mehrjerdi, Alireza Noroozi

**Affiliations:** 1 Iranian National Center for Addiction Studies (INCAS), Tehran University of Medical Sciences, Tehran, IR Iran; 2 Iranian National Center for Addiction Studies (INCAS), School of Advanced Medical Technologies (SAMT), Tehran University of Medical Sciences, Tehran, IR Iran

**Keywords:** Methamphetamine, Blood-Borne Infections, Communicable Diseases, Health, Iran

Recently, Iran has faced a rapidly growing HIV/AIDS problem particularly among injecting drug users (IDUs). A study on IDUs who had been admitted at some DICs in Iran revealed that 20.5% were infected with HIV and 43.4% were infected with HCV (1). Studies on drug injection in Iran have been generally oriented toward opioid users (2) and transmission of blood-borne infection diseases (3). In recent years, a new trend of methamphetamine injection is also observed in the community. Methamphetamine use could contribute to practicing injection, and transmission of blood-borne infection diseases. Studies in developed countries show that as a highly addictive psychostimulant drug, methamphetamine use can increase high-risk sexual behaviors and injection, and the risk of being infected with HIV, hepatitis, and tuberculosis (4-6). Compared to other substances, methamphetamine use is more often linked to increased casual partners and decreased condom use (7, 8), unprotected sex and increased risk for being infected with sexually transmitted infections (STIs) (9, 10), increased involvement in sex trade, needle sharing and failure to clean used needles (5, 11). A study on 139 heterosexual cases who were methamphetamine addicts showed that they reused syringes and shared needles (12). Risky sex among heterosexual and homosexual users of methamphetamine has also contributed to higher rates of hepatitis and HIV infection rates (13, 14). Methamphetamine use also interferes with the efficacy of HIV medications and treatment (15). Methamphetamine is a newly introduced drug in Iran. As a developing country, a recent UNODC world drug report shows that methamphetamine seizures are increasing in Iran (16). Currently, methamphetamine use is observed in emergency departments of psychiatric hospitals (17), among young people in the community (18), opiate-dependent patients who refer to methadone maintenance treatment (19), and IDUs who share syringes (20). In recent years, a harm reduction approach has been rapidly developed for IDUs in Iran. Methadone and buprenorphine maintenance treatment programs have become the main forms of harm reduction programs in the country since 2002 (21) and needle and syringe programs (NSPs) have contributed to a decrease in sharing practices among IDUs (22) but these programs have been implemented for injecting opiate users and it has remained unknown whether the current available interventions could meet the treatment needs of injecting methamphetamine users (IMUs) as a group of psychostimulant users in the country. It should be noted that Iranian IMUs are likely to be at risk for being infected with blood-borne infection diseases but the available information is often of poor quality. This issue may be partly due to the new presence of methamphetamine as a psychostimulant drug in the community. In the other words, in Iran, the investment in treatment of blood-borne infection diseases among IMUs is considerable in recent years. However, the investment in research has been inadequate, making it difficult to assess the epidemics of blood-borne infection diseases among IMUs and evaluate the efficacy of implemented treatment and harm reduction programs. It is possible that a high rate of blood-borne infection diseases has occurred among IMUs in some parts of Iran and has not been recognized yet. As a result, there is a need for conducting epidemiological studies on methamphetamine injection in Iran to investigate the prevalence of methamphetamine injection, the spread of blood-borne infection diseases and control its health implications in all parts of the country. Such studies could contribute to having a more complete nationwide profile of the problem, designing better prevention, treatment and harm reduction programs and improving the quality of current services based on the treatment needs and drug-related characteristics of IMUs. If such studies are not conducted, the healthcare system of Iran will have to prepare itself for long-term consequences of spreading blood-borne infection diseases that are associated with methamphetamine injection in the country. Future studies should be focused on breaking the cycle of blood-borne infection diseases by implementing multidisciplinary interventional approaches to control the spread of these infections among IMUs and to the general population. A study on the feasibility of offering late-night harm reduction services for a group of methamphetamine-using men showed that providing needle exchange, condoms, sexually transmitted infection testing and harm reduction education could decrease the risk of being infected with HIV (23). Drug use prevention and treatment programs that prevent or slow drug injection had profound effects on the HIV and HCV epidemics in other countries (24). Intervention efforts should be expanded progressively in Iran to include comprehensive prevention of blood-borne infection diseases with educational programs, and methamphetamine use therapy, and some therapeutic components such as specific drug-related educational programs must be incorporated for preventing blood-borne infection diseases among IMUs.
